# A Finite Element Analysis on the Effect of Scanning Pattern and Energy on Residual Stress and Deformation in Wire Arc Additive Manufacturing of EH36 Steel

**DOI:** 10.3390/ma16134698

**Published:** 2023-06-29

**Authors:** Muhammad Hassaan Ali, You Sung Han

**Affiliations:** Department of Mechatronics Engineering, Incheon National University, 119 Academy-ro, Yeonsu-gu, Incheon 22012, Republic of Korea

**Keywords:** finite element analysis, material properties at high temperature, residual stress, wire arc additive manufacturing

## Abstract

Wire arc additive manufacturing (WAAM) is a metal additive manufacturing (AM) technique that has a high throughput and has seen a potential interest for replacing currently available subtractive manufacturing techniques. Contrary to other metal AM machines, WAAM rigs can be built using existing welding plants and using welding wire as feedstock, thus, making it a cheap and viable manufacturing technique for a number of industries, such as the maritime industry. However, the effects of AM parameters, such as the scanning pattern and energy, on the residual stress and deformation, are still not completely understood. In this work, a finite element (FE) study has been conducted to understand the influence of different scanning patterns (alternate, in-out, raster and zigzag) and energies on residual stress and warpage. Analyses show that the in-out scanning pattern leads to the highest residual stress, while the zigzag pattern results in the lowest residual stress for all scanning energies considered in this study. Findings in the present study also show that the scanning pattern affects the residual stress and deformation more than does the scanning energy.

## 1. Introduction

Additive manufacturing (AM) is a technology with which nearly net-shaped parts and structures can be produced with high throughput and reduced manufacturing time. Due to this reason, this technology has gained a lot of attention as an alternative to traditional manufacturing processes. With the current advancements in metal AM techniques, metal AM printers have become more readily available to researchers and manufacturers, thus, bridging the gap for rapid prototyping and, ultimately, allowing rapid manufacturing.

In the AM process, parts are built by subsequently stacking many layers of material, where the overall shape of the part depends on the 3D-model. Wire arc additive manufacturing (WAAM) is the one of the most favorable among metal AM techniques [1] and can be used to manufacture large parts which may be several meters long [2]. 

In comparison to other methods in the metal AM category, a WAAM rig is easy to build by fitting an arc-welder to a robotic arm [3]. Since arc welding is the basis of this manufacturing technique, much of the physics resembles a multi-pass arc welding process. Some factors that affect welding, such as the heat source power and welding speed, have a significant impact on the WAAM process as well. Additionally, there are parameters unique to WAAM, such as the scan pattern, hatch size spacing, powder feed rate and cooling time, that must be considered. The thermal history of the parts produced is crucial in determining the final microstructure, part distortion and residual stress, which can lead to problems such as buckling, fatigue, brittle fracture and stress-induced cracking, if not properly managed.

As reported by Liu et al. [4], general 3D printing-filling methods can not be applied to WAAM due to its high deposition rate and high heat inputs. Liu broadly categorized the patterns into linear and contour types, where the linear patterns include the raster, alternate and zigzag paths and the out-in and in-out patterns. The linear patterns, raster and zigzag, are by far the most commonly used WAAM patterns, owing to the simplicity of their implementation and shorter deposition times [5]. The alternate and raster patterns depend upon discontinuous individual lines to form the desired shapes, causing filling inaccuracies and geometrical variations in the final part. Due to this, higher residual stresses are expected in the direction of the deposition, as well as at the start and end positions of the arc [6]. To mitigate this problem, the zigzag pattern combines the individual lines at the end position of a line to the start position of another line, thus, reducing the starts and stops required per deposit [7]. Furthermore, even though other continuous patterns, such as in-out and out-in, which are not linear, do not exhibit longitudinal residual stress patterns, they generally exhibit higher residual stress and warping due to higher accumulation of heat and longer cooling times [8].

Multiple studies have been conducted on the effects of patterns on residual stress and warpage. Li and colleagues [9] used ABAQUS to conduct a thermo-mechanical numerical study to evaluate the impact of AM scanning patterns on residual stress in aluminum alloy 2319. They explored different scan patterns, including zigzag, raster, alternate, in-out spiral and out-in spiral. Cheng et al. [10] also used ABAQUS and compared scan patterns in selective laser melting of In718 and found that 45° inclined line scanning patterns resulted in lower residual stresses compared to other methods. Jia et al. [11], using a commercial finite element software, conducted numerical studies paired with experiments to examine the effects of scanning patterns on Ti-6Al-4V parts made using selective laser melting. They found that a 15° rotated scan strategy produced the lowest level of residual stress. Somashekara et al. [12] studied the effects of scan patterns on residual stress in steel twin-wire welding-based additive manufacturing by using the ANSYS finite element software. The results suggest that raster patterns are recommended for TWAM, but the authors note that the accuracy of the numerical model can be improved by taking into account phase transformations. Wu et al. [8] theoretically and experimentally studied the effects of one spiral and two line patterns on multi-layer WAAM structures of Ti-6Al-4V and In 718. By using the multi-component strain approach, they performed thermo-mechanical simulations in ABAQUS and found that short track length linear patterns displayed the lowest levels of stress, whereas spiral patterns displayed the highest stresses, regardless of the material. Kohler et al. [13] experimentally and through FE simulations using ABAQUS, compared their proposed S-pattern to spiral and meander patterns on Al-4046. They concluded that patterns significantly affect residual stress and warpage in the final built part.

This study is a continuation of our previous work [14], where we studied the effects of patterns of a single layer deposition of EH36 steel parts. Previously, we studied how phase transformations affect the final residual stress and warpage of single layer WAAM parts. In the current study, we examine the impact of different patterns and heat inputs on the residual stress and warpage of multi-layer parts. Comparisons have also been made between single and multi-layer depositions for the same heat source power.

AM scanning pattern and energy have significant effects on the residual stress and deformation of the final part, leading to its durability and life-cycle performance. In the present study, the effects of scanning pattern and energy are investigated using finite element simulations. Four different scanning patterns: alternate, in–out, raster and zigzag scanning strategies, are analyzed. In addition, three different levels of scanning energy: 2300, 2500 and 3000 W, are examined for residual stress and deformation resulting from WAAM using EH36 steel. Analyses on thermal history, residual stress distribution and warpage are performed to determine the optimal AM parameters. Analyses are also conducted to identify relationships between its process properties and product reliability.

## 2. Materials and Methods

Computational techniques such as FEM and Computational Fluid Dynamics, have been extensively employed to analyze complex multi-physical phenomena. Specifically, thermo-mechanical simulations have been utilized to establish a comprehensive relationship between performance characteristics and various process parameters. This study utilized the finite element method (FEM) to examine the impact of deposition patterns on the temperature field, residual stress and warpage while varying deposition power as the process parameter.

The residual stress and deformation of AM products are highly impacted by their thermal history, including temperature distribution and cooling rate; the thermal history of the built part is severely affected by the amount of input heat. The AM process involves localized heat input, leading to a significant temperature gradient throughout the product, causing varying stresses. The study focuses on the impact of the AM scan pattern on thermal history changes, residual stresses and warpage. Four scan patterns—alternate, in-out, raster and zigzag—are analyzed. The alternate, raster and zigzag patterns are line-paths, while the in-out pattern utilizes a contour-path. The alternate and raster patterns are discontinuous, requiring multiple starts and stops, while the zigzag pattern is continuous, but requires precise process control. The in-out pattern follows spiral contours and requires fewer passes but produces excessive thermal gradients, which can lead to void formation. In this study, three levels of heat source power, 3000, 2500 and 2300 W, are also considered to examine the effect of the amount of heat input power on the residual stress and warpage. 

### 2.1. Thermal Analysis

The heat transfer process is modelled using the transient heat conduction equation with a source term. The heat is added to the system by heat conduction between the layers and the substrate, while heat loss occurs due to radiation and convective heat transfer from the layers and substrate to the environment, which is kept at 20 °C. This process can be described as:

(1) $$C_{p}\rho \frac{dT}{dt}=\frac{\partial }{\partial x}\left(\kappa \frac{dT}{dx}\right)+\frac{\partial }{\partial y}\left(\kappa \frac{dT}{dy}\right)+\frac{\partial }{\partial y}\left(\kappa \frac{dT}{dy}\right)+Q,$$

where $C_{p}$ is the heat capacity, ρ is the density, κ is the thermal conductivity, T is the temperature and Q is the heat source term.

Goldak’s [15] double ellipsoidal heat source model has been extensively used to model the wire arc welding process and the WAAM process. This heat source model is defined with the help of two ellipsoids, the front and rear ellipsoids, which are defined by the equations:

(2) $$Q_{f}=\frac{6\sqrt{3}f_{f}Q}{a_{f}bc\pi \sqrt{\pi }}\exp\left(-\frac{3x^{2}}{a_{f}^{2}}-\frac{3y^{2}}{b^{2}}-\frac{3z^{2}}{c^{2}}\right)$$

and

(3) $$Q_{r}=\frac{6\sqrt{3}f_{f}Q}{a_{r}bc\pi \sqrt{\pi }}\exp\left(-\frac{3x^{2}}{a_{r}^{2}}-\frac{3y^{2}}{b^{2}}-\frac{3z^{2}}{c^{2}}\right).$$

The location of the heat source is represented by the spatial coordinates x, y and z, which follow the deposition path. The front and rear lengths of the heat source are represented by $a_{f}$ and $a_{r}$, and the width and depth are represented by b and c, respectively. The energy input is denoted by Q, and the choice of $f_{f}+f_{r}=2$ is based on our previous study [14]. The values for the parameters used in the study can be found in Table 1, which were adjusted to align with previous studies. 

The deposited layer was subject to convection and radiation boundary conditions of 5.7 and 0.2 $W/m^{2}\;K$, respectively. The substrate was assigned an equivalent convection coefficient of 167 $W/m^{2}\;K$, which was chosen to match the temperature profile in Ding’s [16] work. The heat transferred to the environment was compensated in the overall heat transfer by the following equations:

(4) $$q_{convection}=h\left(T-T_{amb}\right)$$

and

(5) $$h_{radiation}=\varepsilon \sigma (T^{4}-T_{amb}^{4})$$

Here, h is the convection coefficient, ε is the radiation coefficient and σ is the Boltzmann constant. $T_{amb}$ is the temperature of the surrounding. 

Other aspects of the study were determined using power-speed correlations from Hu and Qin’s [17] work, and speed of the heat source was set at 10 mm/s.

### 2.2. Mechanical Analysis

The stress analysis is carried out by considering an isotropic material, with the following strain components:

(6) $$\varepsilon^{tot}=\varepsilon^{E}+\varepsilon^{P}+\varepsilon^{Th},$$

where $\varepsilon^{tot}$ is the total strain, $\varepsilon^{E}$ is the elastic strain, $\varepsilon^{P}$ is the plastic strain and $\varepsilon^{Th}$ is the thermal strain. The stress is calculated using the generalized form of Hooke’s Law:

(7) $$\sigma_{ij}=C_{ijkl}^{e}\varepsilon_{kl}^{tot}.$$

$C_{ijkl}^{e}$ is the stiffness matrix of the material being simulated.

For the mechanical boundary conditions, the bottom of the substrate was clamped by constraining it in all directions as done in other works [9,12,16,18].

### 2.3. Material Properties

**Figure 1:**
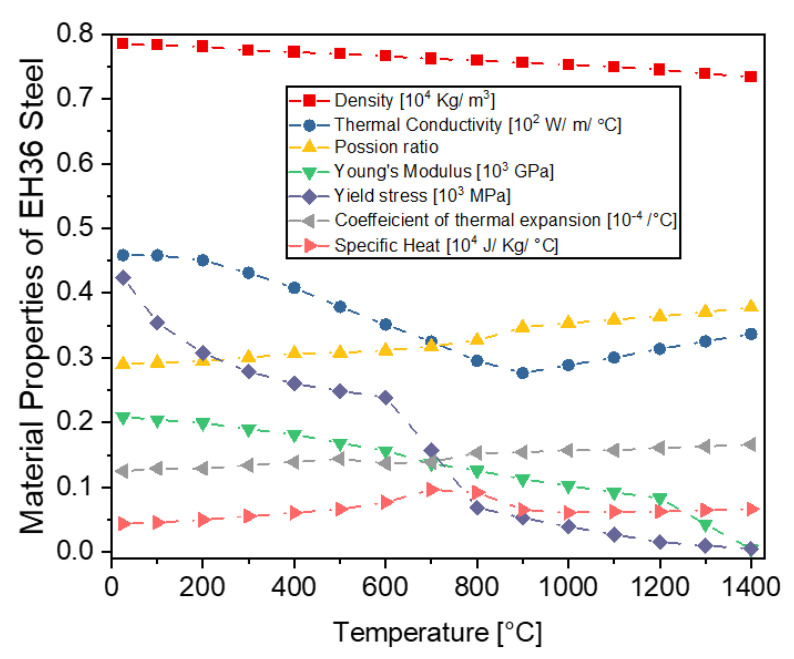
Temperature-dependent material properties of EH36 steel.

Due to its high strength, outstanding welding performance and exceptional toughness at low temperatures, EH36 is a material that is frequently utilized in the shipbuilding sector [19]. Recently, there has been a growing interest in utilizing AM techniques to manufacture parts in the shipbuilding industry. According to Ziolkowski et al. [20], WAAM, Selective Laser Melting (SLM) and Electron Beam Melting (EBM) have been recognized as promising manufacturing techniques that could be effectively applied in the maritime industry. When Wu and colleagues [19] studied the feasibility of fabricating EH36 parts using SLM, they concluded that SLM is indeed capable of producing EH36 parts. Nemani et al. [21] conducted an experimental study comparing mechanical properties and microstructural characteristics of conventionally manufactured EH36 steel plates to additively manufactured EH36 steel plates, in which, they concluded that water quenched WAAM plates exhibit improved mechanical properties. EH36 steel has been chosen for the study’s substrate, wire deposition and WAAM. This choice has been taken in anticipation of future AM use in the marine and shipbuilding sectors. The chemical makeup of EH36 (in weight percent) is: 0.14% C, 1.47% Mn, 0.28% Si, 0.05% Cr, 0.3% Ni, 0.01% Mo, 0.002% S, 0.04% Al, 0.03% V, 0.03% Nb, 0.2% Cu, 0.02% P and the balance is Fe. The temperature dependent material properties are shown in Figure 1 [22]. The latent heat is 251,400 J/Kg and the solidus and liquidus temperatures are 1465.1 °C and 1522.5 °C, respectively. The values of hardening coefficients, H(ε^pl^,T), used in this work are listed in Table 2. 

As mentioned above, the current study is a continuation of our previous study [14]. Material parameters used in this work are the same as in the previous work. The simulation results using parameters, such as phase transformation, temperature history and distribution, etc., were compared with experimental measurements for the purpose of validation.

**Figure 2:**
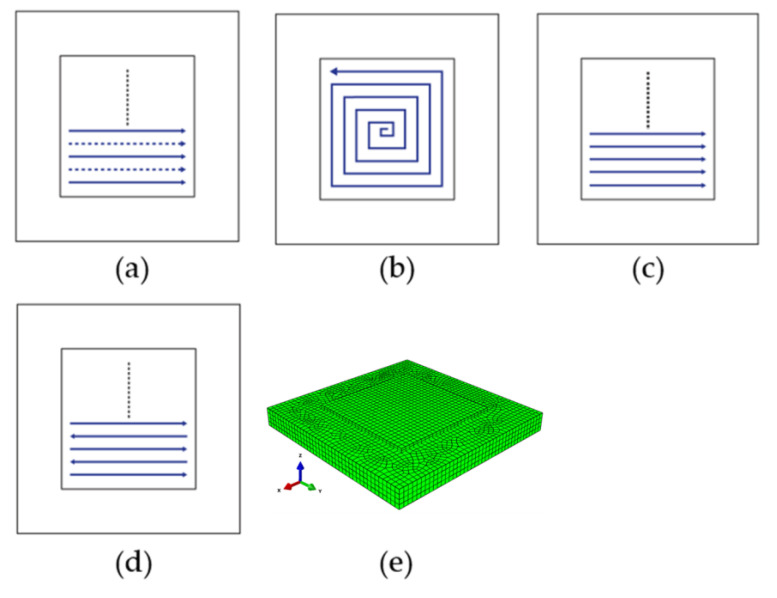
Deposition patterns analyzed (**a**) alternate, (**b**) in–out, (**c**) raster and (**d**) zigzag. The FE model (**e**) used in the present work.

In this work, four scanning patterns are investigated as illustrated in Figure 2a–d: alternate, in-out spiral, raster and zigzag. The next section has further information about the deposition pattern. As seen in Figure 2e, the substrate has dimensions of 200 mm × 200 mm × 20 mm and two deposition layers of 120 mm × 120 mm × 2.3 mm, each. A total of 8248 elements make-up the geometry, of which, 1152 are in the deposition layer with 576 elements in each layer and 7096 in the substrate. In this work, a sequentially linked thermo-mechanical analysis was carried out where all the properties were constant except the power of the heat source, which varied as follows: 2300, 2500 and 3000 W, and with the four deposition patterns for each of these power levels. Thus, the total number of sets of thermo-mechanical simulations is 12.

The simulations were performed using the commercial FE software ABAQUS 2019 (Dassault Systèmes). Linear heat-transfer brick elements with 8-nodes each, DC3D8, were used for the thermal simulation, while C3D8 8-node linear brick elements were used for the mechanical part of the simulations. The AM-plugin, which is a module built specifically for AM simulations, was used for our simulations. This works by using the progressive element activation technique for material deposition. Heat source definitions are also built-in to the AM-plugin, thus, no UMAT, such as DFLUX, was needed to be used for the heat source.

### 2.4. Deposition Patterns

Thermal histories and cooling rates strongly influence residual stresses and part deformation of additively manufactured parts. The varying temperature gradients and localized heat inputs during different deposition processes cause varying levels of stress throughout the built part. In this research, we examine the effect of varying source power on the different deposition patterns, their residual stresses and warpage of the final part. Four distinct patterns have been analyzed: alternate, in-out, raster and zigzag patterns, as shown in Figure 2. The alternate, raster and zigzag patterns are linear patterns while the in-out pattern is in the form of a spiral contour-path. Discontinuous patterns such as alternate and raster patterns require multiple starts and stops, while the continuous zigzag pattern reduces the number of passes but requires precise control of process parameters. The spiral contour pattern of in-out allows for fewer passes, but also results in excessive thermal gradients at the start and end of the scan, which can lead to the formation of voids.

## 3. Results and Discussions

We first compare the temperature profile of the current results for a two-layer model with our previous results of a single layer model [14]. Furthermore, since all residual stresses generated in our current simulation are due to the heat added to the system, it is important to analyze how the temperature history of the different patterns varies across the model, for the different levels of heat source power. Finally, the residual stresses and warpage of the different patterns are compared.

### 3.1. Temperature Analysis

**Figure 3:**
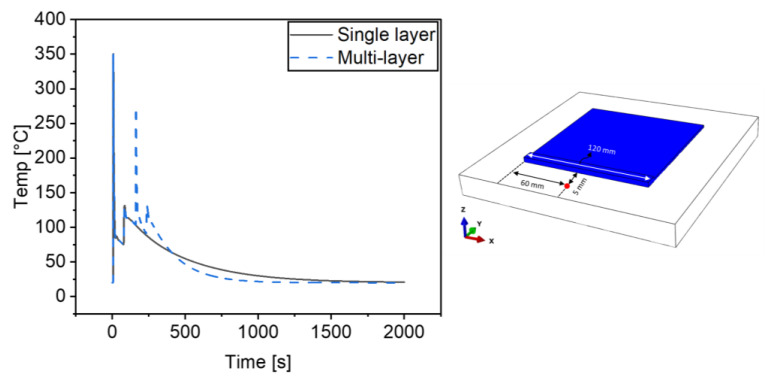
Verification of the current temperature calculations at the point 5 mm away from the deposition path compared with single deposition at 3000 W.

The temperature profile in our previous study was verified using the results from the analysis of Ding et al. [16]; therefore, for this study, we have compared the results from our previous study. We used the temperature profile from the alternate case of the previous and current study, which had a heat source power of 3000 W. The point of investigation was located 5 mm away from the path of the deposition, as shown in Figure 3. The Figure 3 also shows the thermal profile for a two-layer simulation, compared to the single layer simulation from our previous study [14]. Since the thermal properties used in both the simulations are the same, the temperature profile for the first layer is also the same; however, the temperature profile for the second layer has a much steeper descent. This can be attributed to two reasons, the first is that the point of analysis is further away from the heat source than it was for the first layer. The second reason is that the surface area has now increased due the addition of a second layer, and this increases the rate of heat loss to the environment.

**Figure 4:**
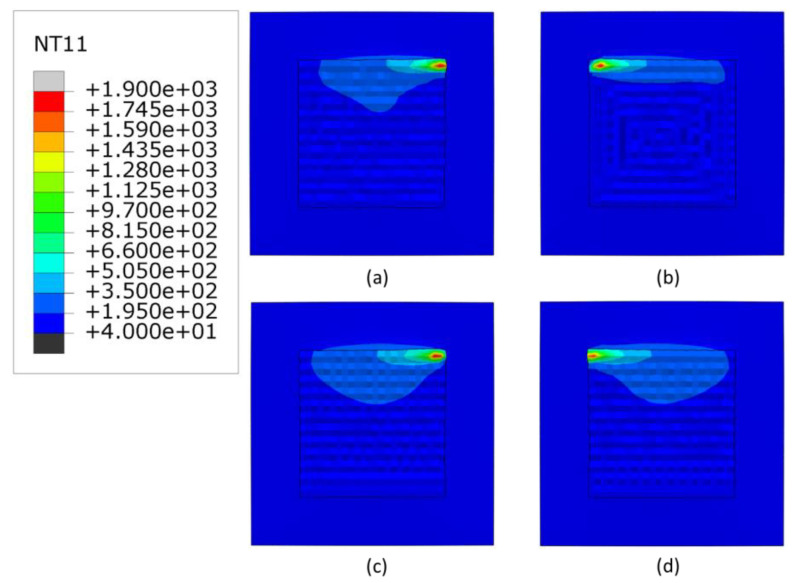
The temperature distributions of all patterns considered at the end of deposition process, when heat source power is 3000 W. (**a**) alternate, (**b**) in–out, (**c**) raster, (**d**) zigzag (Top view, unit: °C).

**Figure 5:**
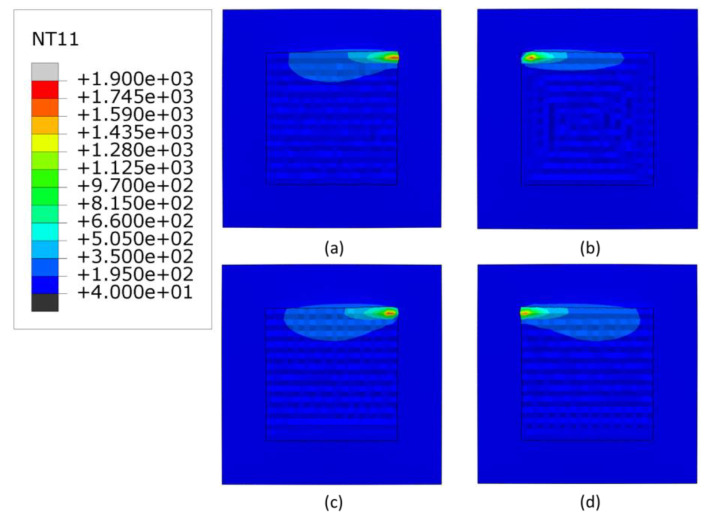
The temperature distributions of all patterns considered at the end of deposition process when heat source power is 2500 W. (**a**) alternate, (**b**) in–out, (**c**) raster, (**d**) zigzag (Top view, unit: °C).

**Figure 6:**
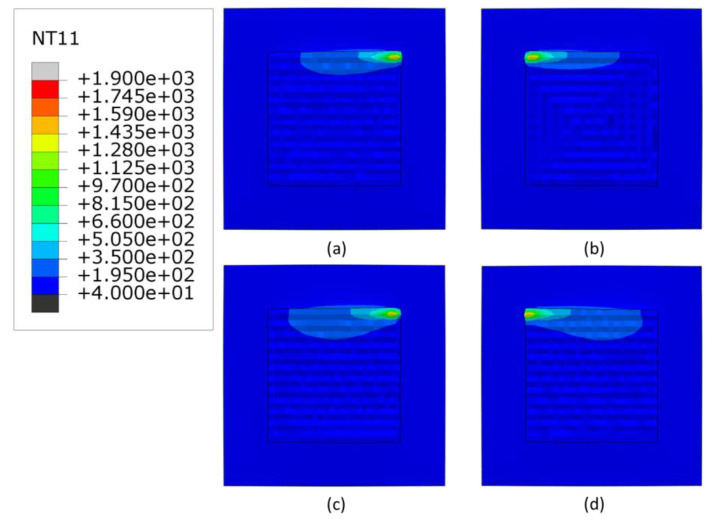
The temperature distributions of all patterns considered at the end of deposition process when heat source power is 2300 W. (**a**) alternate, (**b**) in–out, (**c**) raster, (**d**) zigzag (Top view, unit: °C).

Figure 4, Figure 5 and Figure 6 show the temperature contours at the end of the deposition process for 3000, 2500 and 2300 W, respectively. As expected, with higher deposition power, the meltpool temperature increases. The minimum temperature of the substrate is higher (40 °C) than the room temperature, due to the accumulated heat from the overall scans. Generally, the raster scans have higher heat retention than other scanning patterns, whereas the in-out scanning pattern has the lowest heat retention. Among the linear scanning patterns, the alternate scanning pattern has the lowest heat retention due to its discontinuous nature.

### 3.2. Stress Analysis

**Figure 7:**
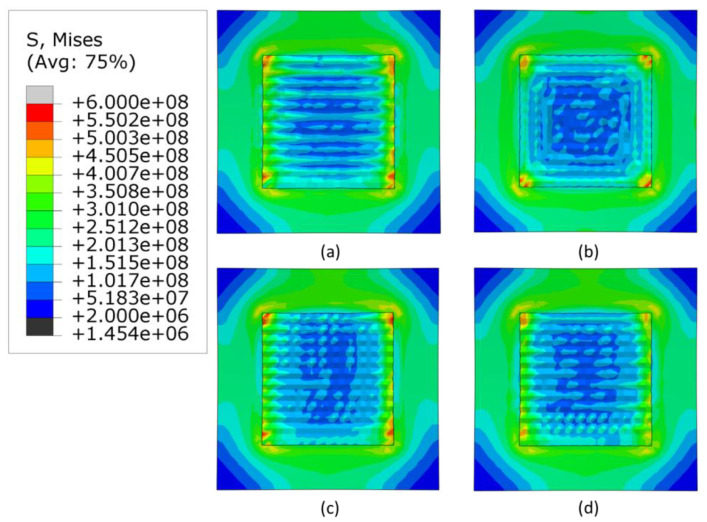
Mises stress distribution of different deposition patterns at the end of the simulation, when heat source power is 3000 W, (top view, unit: Pa); (**a**) alternate, (**b**) in–out, (**c**) raster, (**d**) zigzag.

**Figure 8:**
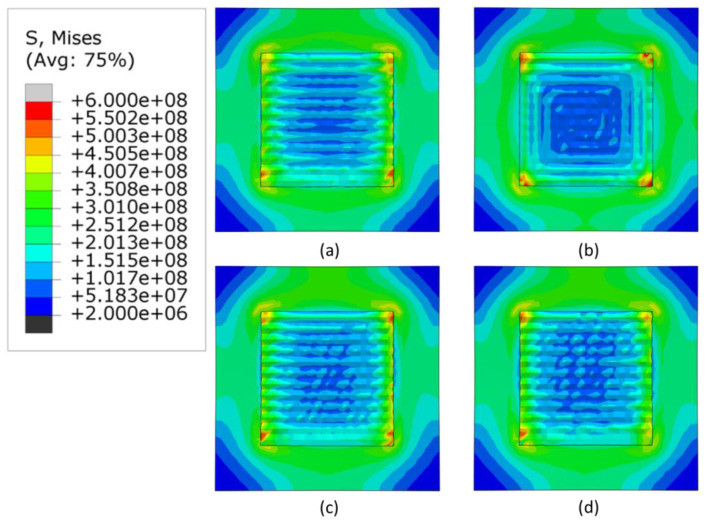
Mises stress distribution of different deposition patterns at the end of the simulation, when heat source power is 2500 W, (top view, unit: Pa); (**a**) alternate, (**b**) in–out, (**c**) raster, (**d**) zigzag.

**Figure 9:**
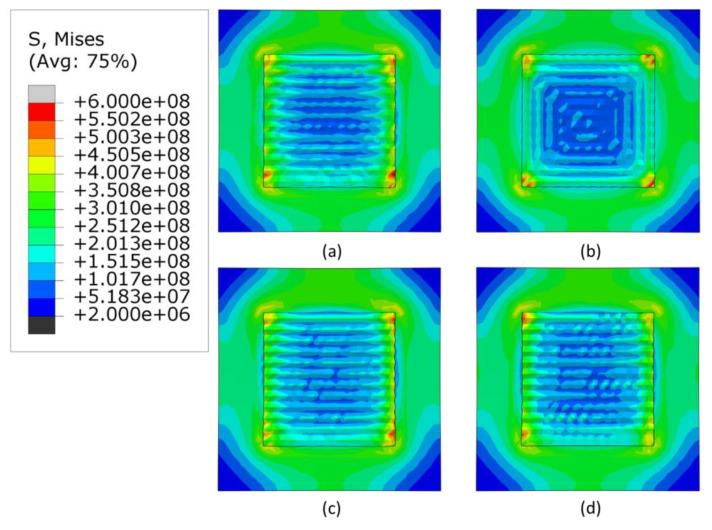
Mises stress distribution of different deposition patterns at the end of the simulation, when heat source power is 2300 W, (top view, unit: Pa); (**a**) alternate, (**b**) in–out, (**c**) raster, (**d**) zigzag.

The Figure 7, Figure 8 and Figure 9 show the stress distribution after cooling and removal of clamps when the heat source applied is 3000, 2500 W and 2300 W, respectively. For all levels of power, the highest stresses are concentrated at the corners of the deposition area and the lowest stresses are concentrated at the center of the deposited layers and the corners of the substrate. The alternate pattern shows negligible changes in stress generated by the three heat sources. For the in-out pattern, the minimum stresses decrease significantly in the deposited area. Meanwhile, the raster and zigzag patterns have lowest stresses in deposition area, which progressively increase for lower levels of heat source power.

**Figure 10:**
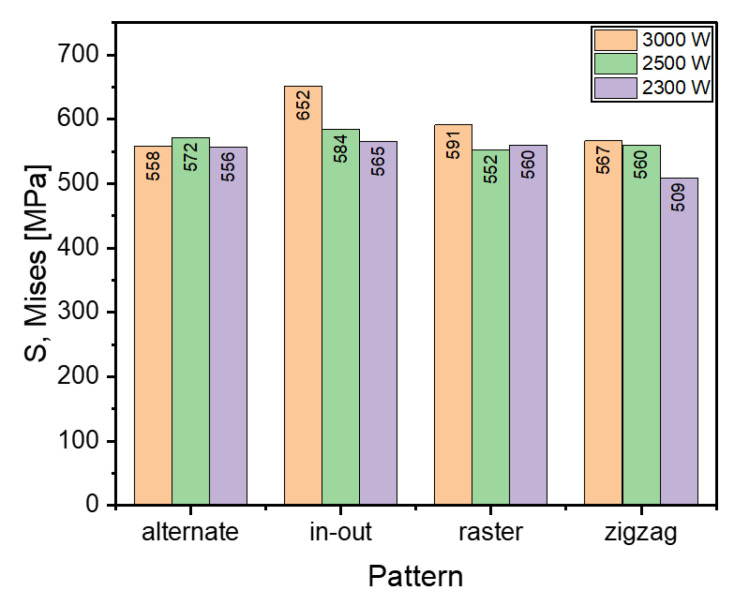
Comparison of maximum Mises stress of different deposition patterns at the end of the simulation when heat source power is 3000, 2500 and 2300 W.

Figure 10 shows that the in-out pattern has the highest maximum stress for all cases. Among all power levels and patterns, the lowest stress is generated by the lowest power in the zigzag pattern. When the heat source was 3000 W, the highest maximum stress was generated in the in-out pattern and the lowest in the alternate pattern. This is inconsistent with the other heat source powers, in which, the lowest maximum stress was in the zigzag pattern. The alternate and raster patterns do not exhibit the trend of increasing stress with increaing heat source power. The reason behind this may be due to the discontinuous nature of these patterns. However, the overall differences in values for the alternate and raster patterns are quite small.

### 3.3. Warpage Analysis

**Figure 11:**
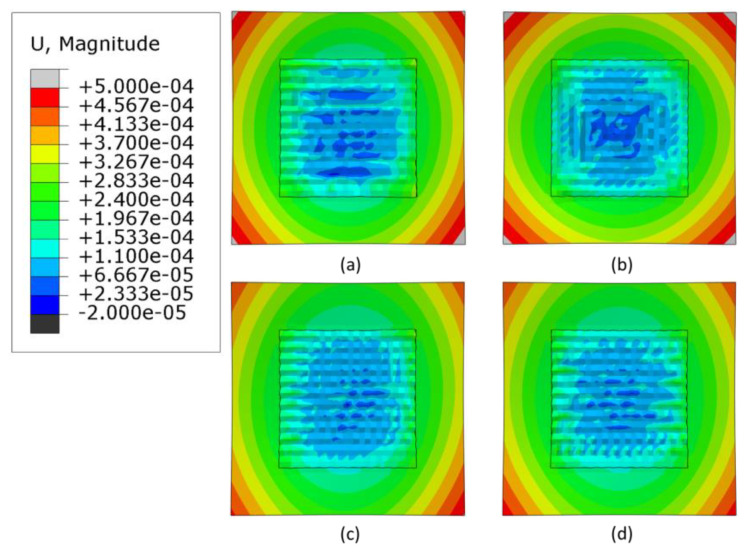
Deformation distribution of different deposition patterns at the end of the simulation, when heat source power is 3000 W, (top view, unit: m); (**a**) alternate, (**b**) in–out, (**c**) raster, (**d**) zigzag.

**Figure 12:**
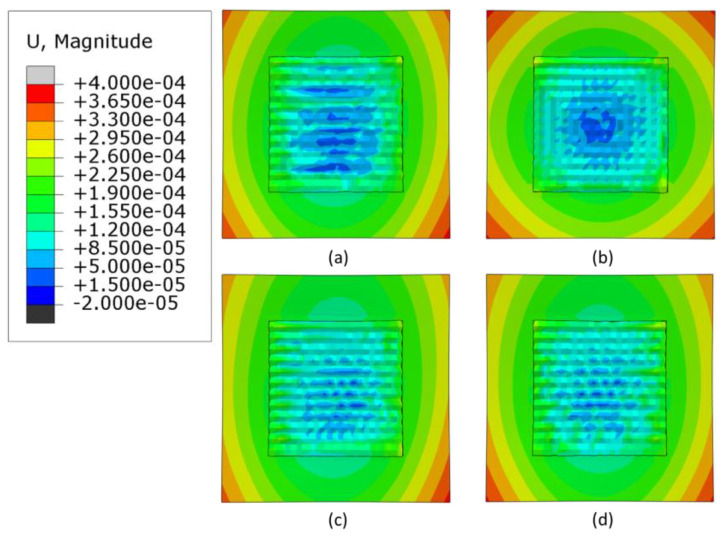
Deformation distribution of different deposition patterns at the end of the simulation, when heat source power is 2500 W, (top view, unit: m); (**a**) alternate, (**b**) in–out, (**c**) raster, (**d**) zigzag.

**Figure 13:**
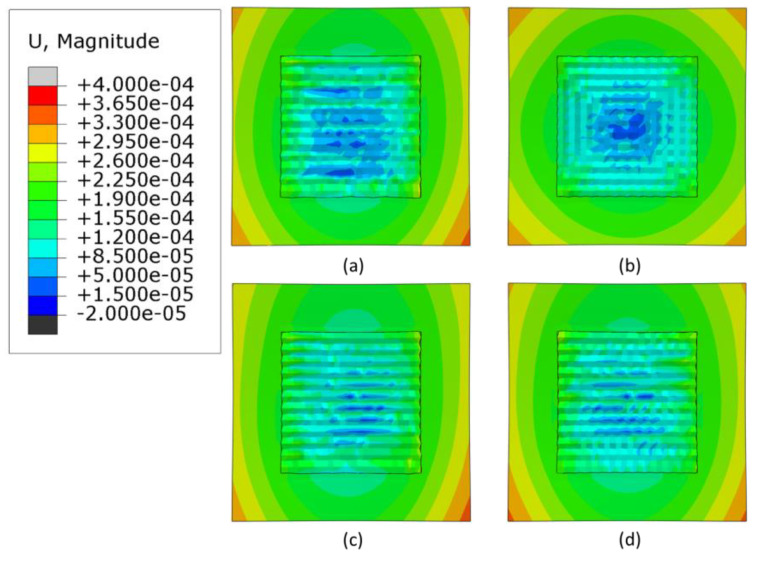
Deformation distribution of different deposition patterns at the end of the simulation, when heat source power is 2300 W, (top view, unit: m); (**a**) alternate, (**b**) in–out, (**c**) raster, (**d**) zigzag.

**Figure 14:**
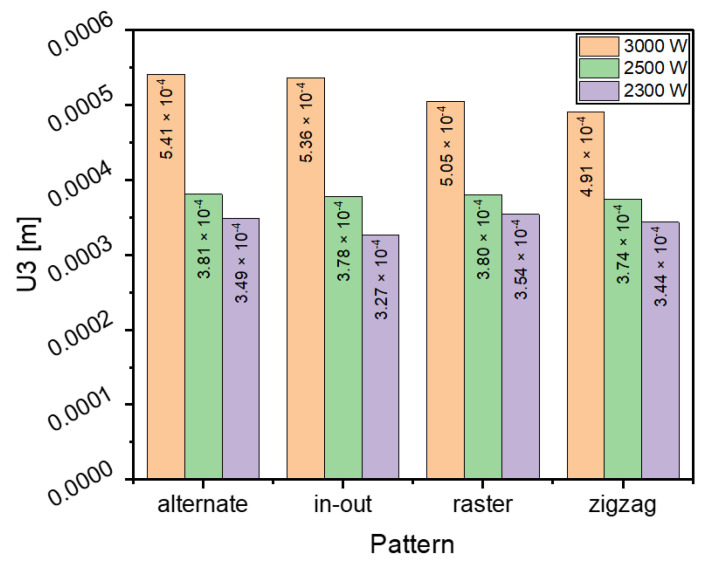
Comparison of maximum deflection of different deposition patterns at the end of the simulation when heat source power is 3000, 2500 and 2300 W.

Warpage is an important factor to consider when manufacturing AM parts; if it is not compensated for, a distorted manufactured part may result. Figure 11, Figure 12 and Figure 13 shows contour plots from the z-direction, which is also the build direction. In general, the plots show that the part distorts inwards into the page at the center where the layers have been deposited, while the corners of the substrate distort out of the page. The magnitude of distortion is higher when the heat source power is 3000 W, which is followed by 2500 W and then 2300 W. In general, an increased heat source power causes greater distortion. This is evident from Figure 14, which shows the maximum warpage for the three heat source power levels of 3000, 2500 and 2300 W. Between 3000 W and 2500 W, there is a difference of 500 W, and between 2500 W and 2300 W there is a difference of 200 W, thus, the largest differences in warpage resulted in the comparison between 3000 W and 2300 W.

### 3.4. Difference between Single Layer and Multi-Layer Results

#### 3.4.1. Stress

**Figure 15:**
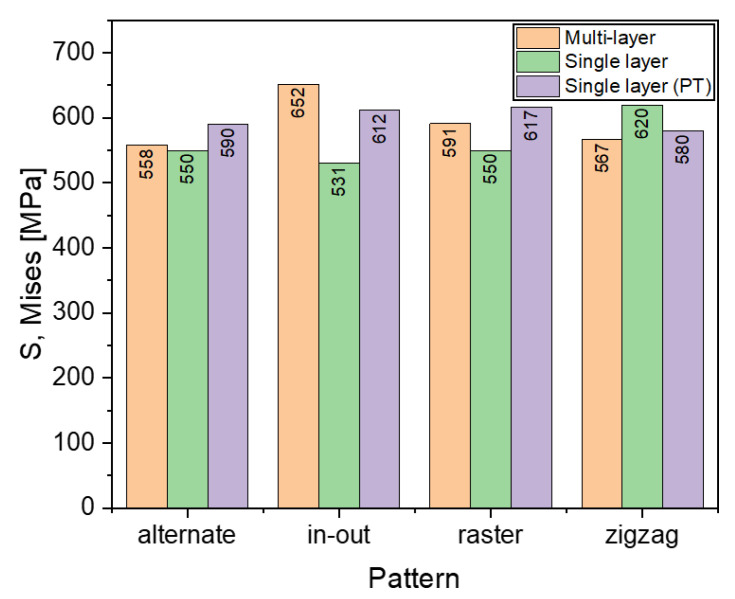
Comparison of maximum Mises stress of different deposition patterns at the end of the simulation for single layer (with and without phase transformations (PT)) and multilayer depositions when heat source power is 3000 W.

Comparisons in stresses and warpage between a single layer deposition and a multi-layer deposition with two layers are discussed in this section. It can be seen from Figure 15 that the continuous patterns, in-out and zigzag, have significant differences between the stresses, whereas the discontinuous line patterns, alternate and raster, do not show significant differences. The alternate pattern has the least difference between the single layer and multi-layer variants, while the in-out pattern has the greatest difference between the layering strategies. The single layer results when phase transformations (PT)_are considered, show results comparable to the multi-layer results.

#### 3.4.2. Warpage

**Figure 16:**
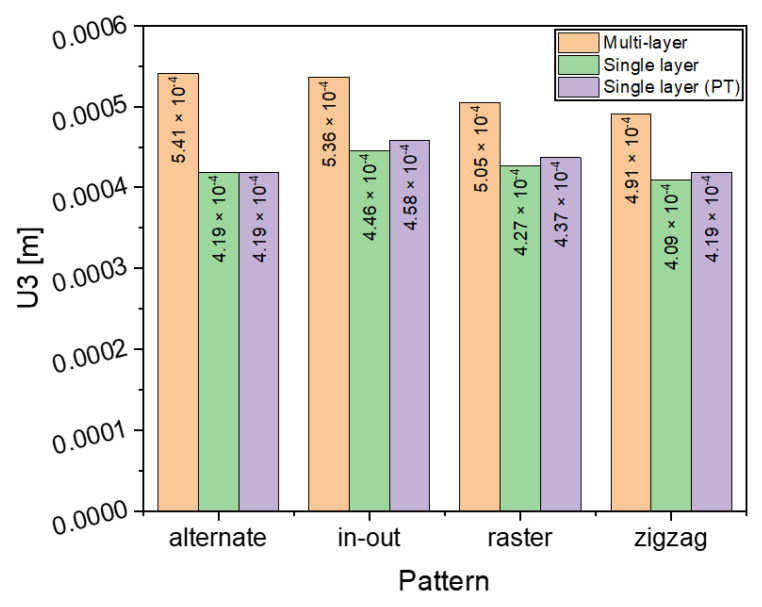
Comparison of maximum deflection of different deposition patterns at the end of the simulation for single layer (with and without phase transformations (PT)) and multilayer depositions when heat source power is 3000 W.

The differences in warpage for the multi-layer and single layers are highlighted in Figure 16, which shows that additional layers considerably increase the distortion of the final part. However, the Figure 16 also shows that there is no significant change in the warpage across the different patterns. The warpage remains almost the same for the two single layer cases whether PT are considered or not, whereas for the multi-layer the warpage increases with the increase of a new layer.

## 4. Conclusions

In this work, FE simulations were performed to examine the effect of AM scanning pattern and energy on the residual stress and deformation in EH36 steel. Sequentially coupled thermo-mechanical simulations were performed to simulate the WAAM multiple layer depositions with four different scanning strategies (alternate, in–out, raster and zigzag scanning) and three different heat source energies (2300, 2500 and 3000 W). The major findings are as follows:

1. Analyses show that the in-out scanning pattern leads to the highest residual stress (652 MPa) while the zigzag pattern results in the lowest residual stress (567 MPa) among all scanning energies considered in this study. The difference is up to 15%. This trend results from the discontinuous nature of the pattern.
2. The difference between the highest and the lowest stress across patterns decreases with lower heat source power. When the heat source power is 3000 W, the difference is 85 MPa, while it decreases to 56 MPa. It should be noted that the influence of heat source energy on the residual stress decreases for all cases considered in this study.
3. Warpage/distortion is proportional to the power of the heat source, regardless of the pattern. The deflection for 3000 W heat source energy is calculated as 0.41–0.54 mm; it decreases to 0.33–0.35 mm, depending on the scanning type.
4. With added layers, residual stress increases significantly for the in-out pattern (from 531 MPa to 652 MPa) and slightly increases for the raster pattern (from 550 MPa to 591 MPa).
5. The value of the highest warpage increases with the addition of a new layer. It increases by 23% in the alternate pattern, and by 15–16% for other scanning patterns considered in this study.
6. Findings in the present study also show that the scanning pattern affects the residual stress and deformation more than does the scanning energy.

The present work can be applied to determination of process parameters for other materials used in WAAM, such as titanium and aluminum alloys. Currently, there is a study of realistic AM conditions for multi-pass and multi-layer WAAM fabrications, along with collaborative experimental research. The numerical model and the simulation setups in this work and the phase transformation calculation in the authors’ previous work could be extended to other AM processes such as powder bed fusion or electron beam melting.

## Figures and Tables

**Table 1:** The parameters for the double ellipsoidal heat source model used in the present study.

$a_{f}$ (mm)	$a_{r}$ (mm)	b (mm)	c (mm)	$f_{f}$	$f_{f}$
2	6	2.5	2.3	0.6	1.4

**Table 2:** The hardening coefficient, H(ε^pl^,T), used in the present study.

ε^pl^	H (MPa) (T = 20 °C)	H (MPa) (T = 700 °C)	H (MPa) (T = 1300 °C)
0.0	0	0	0
0.1	290	200	8.75

## Data Availability

Not applicable.
